# Quality of dispatch‐assisted cardiopulmonary resuscitation by lay rescuers following a standard protocol in Japan: an observational simulation study

**DOI:** 10.1002/ams2.315

**Published:** 2017-10-11

**Authors:** Hideki Asai, Hidetada Fukushima, Francesco Bolstad, Kazuo Okuchi

**Affiliations:** ^1^ Department of Emergency and Critical Care Medicine Nara Medical University Kashihara Nara Japan; ^2^ Department of Clinical English Nara Medical University Kashihara Nara Japan

**Keywords:** Cardiac arrest, cardiopulmonary resuscitation, emergency medical services, prehospital care/medical control, simulation

## Abstract

**Aim:**

Bystander cardiopulmonary resuscitation (CPR) is essential for improving the outcomes of sudden cardiac arrest patients. It has been reported that dispatch‐assisted CPR (DACPR) accounts for more than half of the incidence of CPR undertaken by bystanders. Its quality, however, can be suboptimal. We aimed to measure the quality of DACPR using a simulation study.

**Methods:**

We recruited laypersons at a shopping mall and measured the quality of CPR carried out in our simulation. Dispatchers provided instruction in accordance with the standard DACPR protocol in Japan.

**Results:**

Twenty‐three laypersons (13 with CPR training experience within the past 2 years and 10 with no training experience) participated in this study. The median chest compression rate and depth were 106/min and 33 mm, respectively. The median time interval from placing the 119 call to the start of chest compressions was 119 s. No significant difference was found between the groups with and without training experience. However, subjects with training experience more frequently placed their hands correctly on the manikin (84.6% versus 40.0%; *P* = 0.026). Twelve participants (52.2%, seven in trained and five in untrained group) interrupted chest compressions for 3–18 s, because dispatchers asked if the patient started breathing or moving.

**Conclusion:**

This current simulation study showed that the quality of DACPR carried out by lay rescuers can be less than optimal in terms of depth, hand placement, and minimization of pauses. Further studies are required to explore better DACPR instruction methods to help lay rescuers perform CPR with optimal quality.

## Introduction

Sudden cardiac arrest (CA) is a leading cause of death in industrialized nations and effective bystander cardiopulmonary resuscitation (CPR) is essential to increase patients’ chance of survival from out‐of‐hospital sudden CA.^1^, ^2^, ^3^ The rate of bystander CPR, however, generally remains low in most communities.^2^, ^4^, ^5^ Thus emergency medical service (EMS) dispatchers who take emergency calls may instruct callers to perform CPR.^6^ Bystander CPR undertaken by lay rescuers under dispatch instruction is called dispatch‐assisted CPR (DACPR).^4^, ^7^, ^8^ It has been reported that DACPR can double the rate of bystander CPR and is associated with a better outcome for sudden CA victims.^9^ However, in terms of the quality of CPR performed by lay rescuers, simulation studies have shown it is generally low^10^, ^11^, ^12^ and the quality of DACPR can also be suboptimal in real cardiac arrest cases. Even in lay rescuers with CPR training experience, performance can be poor as the skills and knowledge deteriorate soon after training.^13^ These lay rescuers, however, are still the best candidates to perform DACPR until EMS personal arrive and dispatchers should understand how they perform CPR. In this study, we hypothesized that the quality of DACPR performed by lay rescuers is suboptimal. To test this hypothesis, we undertook a study to simulate lay rescuers encountering a CA situation, and observed how they perform CPR under EMS dispatch instruction.

## Method

### Study design

This study was approved by the ethics committee of Nara Medical University (Kashihara, Japan). We conducted an observational simulation on non‐health‐care providers to observe how lay rescuers perform CPR under EMS dispatch instructions. The primary outcome of this study was to measure the performance of DACPR by lay rescuers in terms of chest compression quality as well as its time course. The secondary outcome was to observe the effect of CPR training experience on DACPR. We compared the quality of DACPR between lay rescuers with and without CPR training experience. We recruited participants at a shopping mall and all participants were informed about the purpose of this study and written consent was obtained from each. Each subject was offered a $10 value gift card as an incentive for participation in the study.

### Dispatch‐assisted CPR simulation

**Figure 1:**
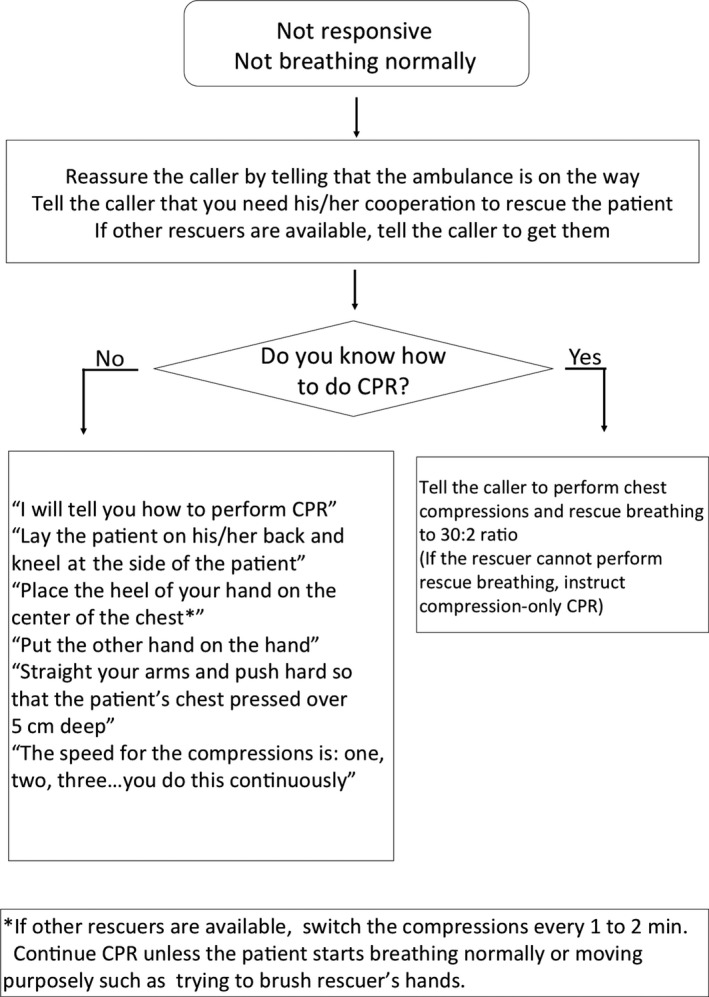
Dispatch‐assisted CPR instruction protocol used in this simulation study.

In this simulation, participants performed a single rescuer scenario in a small room. In this room, there was a manikin (Resusci Anne QCPR; Laerdal, Norway) and a cordless extension phone on the hard surface floor. Neither an automated external defibrillator nor other rescuers were available in this simulation. After being given a list of simple introductions for this simulation, participants entered the room and performed CPR under instruction by off‐duty EMS dispatchers. Only EMS dispatchers with at least 1 year of experience took part in this simulation and provided CPR instruction by telephone from a different room. Dispatchers were instructed not to ask the address, not to instruct the participant to perform rescue breathing, and were strictly instructed to tell the participant to activate the speaker phone function and continue chest compression for 2 min. Dispatchers provided CPR instruction along with the recommended protocol from the Ministry of Internal Affairs and Communications Fire and Disaster Management Agency (Fig. 1).^14^

### Data collection

When obtaining the study consent, we collected sex and age data for the study participants. We also asked the study participants whether they had CPR training experience.

Data for chest compression performance (mean depth [mm], mean rate [compressions per min (cpm)], and correct hand position [%]) were collected through the Laerdal Resusci Anne QCPR. Each simulation was recorded by video cameras (HDR‐AS200V; Sony, Japan). Abdominal hand placement was determined by video camera review with two study investigators as well as the Laerdal QCPR report. Interruption of compressions (s) was also measured by these two investigators using the video camera recordings.

Data regarding the time intervals from the start of the 119 call to the identification of the need for CPR (t1), to the start of CPR instruction (t2), and to the start of chest compressions (t3) was measured afterwards by two researchers.

### Statistics

Continuous variables were described as median and interquartile range and categorical variables were described as numerals (percentages). These variables were compared between the group with or without CPR training experience. We used the Mann–Whitney *U*‐test for continuous variables. Categorical variables were compared between groups by either a χ^2^‐test or Fischer's exact test. Two‐tailed *P*‐values <0.05 were considered significant. Data analysis was carried out spss version 22.0 (SPSS, Chicago, IL. USA).

## Results

Twenty‐three participants were recruited at the shopping mall in this study. The majority of the participants were female (19/23 [82.6%]). Table 1 shows the characteristics of the participants. Among them, thirteen participants had previous CPR training two or more years before (trained group).

**Table 1:** Characteristics of participants

	Total (*n* = 23)	Trained group (*n* = 13)	Untrained group (*n* = 10)	*P* values
Female, *n* (%)	19 (82.6)	13 (100)	6 (60)	0.024^a^
Age brackets				0.576
20–29 y.o., *n* (%)	4 (17.4)	3 (23)	1 (10)	
30–39 y.o., *n* (%)	12 (52.2)	7 (54)	5 (50)	
40–49 y.o., *n* (%)	7 (30.4)	3 (23)	4 (40)	
How long since last CPR training?				–
2–3 years before, *n* (%)	–	4 (31)	–	
3–5 years before, *n* (%)	–	2 (15)	–	
>5 years before, *n* (%)	–	7 (54)	–	

a Fischer's exact test.

Table 2 shows the quality of the chest compression by study participants. The median chest compression rate and depth were 106 cpm and 33 mm, respectively. These variables were similar between the trained group and the untrained group (106 cpm versus 105.5 cpm, *P* = 0.313, and 33 mm versus 33 mm, *P* = 0.193, respectively). Fifteen subjects (15/23 [65.2%]) placed their hands correctly on the center of the manikin's chest. Correct Hand placement was more frequent in the trained group and subjects in the untrained group tended to do abdominal hand placements (84.6% versus 40.0% *P* = 0.026). Twelve participants (52.2%, seven in trained and five in untrained group) interrupted 2‐min chest compressions and observed the simulator when dispatchers asked if the patient started breathing, moving or facial color change. The period of interruption was a minimum of 3 s and a maximum of 18 s.

**Table 2:** Quality of chest compressions

	Total (*n* = 23)	Trained group (*n* = 13)	Untrained group (*n* = 10)	*P* values
Compression Rate, cpm, median (IQR)	106 (93–108)	106 (91.5–110)	105.5 (100.5–107)	0.313
Compression Depths, mm, median (IQR)	33 (25–40.5)	33 (22.5–40.5)	33 (27.5–49)	0.193
Correct hand position, *n* (%)	15 (65.2)	11 (84.6)	4 (40.0)	0.026^a^
Chest compressions Interruption, *n* (%)	12 (52.2)	7 (53.3)	5 (50.0)	0.855
Range of chest compressions Interruption, s, min‐max	3–18	3–18	5–9	0.722

IQR, interquartile range; cpm, compressions per minute.

a Fischer's exact test.

The key time intervals of each step of DACPR are shown in Table 3. The median time intervals from the 119 call: to recognition of cardiac arrests by the dispatcher (t1), to the start of CPR instruction (t2), and to the start of chest compressions (t3) were 48s, 81s, and 119s, respectively. These time intervals were also similar in the two groups.

**Table 3:** Time intervals of DACPR process

	Total (*n* = 23)	Trained groups (*n* = 13)	Untrained group (*n* = 10)	*P* values
Time from 119 call to dispatcher recognition of CA: t1, s, median (IQR)	48 (47–71)	61 (49–76)	49 (47–85)	0.170
Time from 119 call to start of dispatch‐instruction for CPR: t2, s, median (IQR)	81 (74–112)	97 (80–122)	80 (72–115)	0.098
Time from 119 call to start of the chest compression: t3, s, median (IQR)	119 (112–150)	130 (116–169)	123 (109–152)	0.164

IQR, interquartile range; CA, cardiac arrest; CPR, cardiopulmonary resuscitation.

## Discussion

In this simulation study, we found that the quality of DACPR by lay rescuers was suboptimal in terms of compression depth. When comparing participants with CPR training experience to those without training experience, the quality of CPR was similar except for the correct hand position.

DACPR accounts for more than half of BCPR before EMS arrival and is associated with improved outcomes.^7^, ^15^ Its quality, however, can often be suboptimal.^10^, ^11^, ^12^, ^16^ Investigating how lay rescuers perform chest compressions under standard dispatch instruction for CPR and exploring better DACPR instruction to improve the quality of CPR are essential to improve survival with favorable neurological outcomes among sudden CA patients.

Studies have shown that CPR with optimal chest compression depth is associated with better outcomes, but in real life the depth achieved is rarely the recommended 5 cm.^17^, ^18^ Vadeboncoeur *et al*.^19^ reported that optimal chest compression depth can increase the odds for survival by each 5 mm. And yet, several DACPR simulation studies have reported that the depth of chest compressions by lay rescuers rarely achieves the recommended depth.^10^, ^11^, ^16^ Supporting lay rescuers to perform optimal chest compression is challenging. Several studies have reported that giving simple instruction to “Push as hard as you can” was better than giving instruction “Push deep more than 5 cm,” but even with this simple instruction, the depth was still suboptimal.^10^, ^11^ In this study, dispatchers provided the standard protocol provided by the Ministry of Internal Affairs and Communications Fire and Disaster Management Agency^14^: “push hard enough so that the patient's chest compresses more than 5 cm.” Our current study showed that this standard instruction resulted in suboptimal chest compression depth and changes need to be considered for optimal DACPR instruction. It should perhaps be expected that the longer lay rescuers perform chest compressions, the poorer the quality of CPR becomes, but this study showed that the quality can be poor even for the first 2 min. Different approaches may be needed to help rescuers perform chest compressions to the optimal depth.

The rate of chest compressions, on the other hand, were within optimal range around 100 compressions per minute. Dispatchers were able to guide the compression rate by counting numbers and this method was able to facilitate the optimal chest compression rate.

One issue that needs to be addressed is the instruction for correct hand positioning. In this study, eight lay rescuers performed CPR with abdominal hand placement. Instructions for hand placement have been changed throughout the guidelines updates^20^ and have resulted in a significant rate of incorrect hand placement.^21^, ^22^ Birkenes *et al*. observed that lay people understood the center of the chest to be the same as the center of the torso^20^ and nearly half of their study participants who learned CPR even 6–9 months ago placed their hands too low.^21^ Guidelines claim that CPR by lay rescuers is quite safe even for victims who are not in CA.^23^, ^24^ Since depth of chest compressions by lay rescuers is usually suboptimal, this incorrect hand placement would rarely damage abdominal organs such as the liver.^25^ This abdominal hand placement, however, can be harmful if lay rescuers perform hard chest compressions to achieve optimal depth. Dispatcher instructions have mainly focused on CPR technique, but not practical preparations for CPR. The instruction for correct hand position, however, is another key component of EMS dispatch instruction for CPR, and this needs to be investigated. In this regard, Birkenes *et al*.^20^ tried a unique instruction for hand placement to avoid abdominal compression; instructing rescuers to straighten the patient's arm close to the rescuers out from the patient's body and sit astride the arm. This unique technique, however, has not been validated in a large study population. Safe and secure instructions for hand placement still remains to be explored.

Another issue which needs addressing is the interruption of chest compressions. Twelve participants (52.2%) interrupted chest compressions for a minimum of 3 s and a maximum of 18 s, because some dispatchers asked participants if the patients showed any change such as breathing, moving or facial color. Participants observed the simulator when they were asked. According to the standard protocol for DACPR, dispatchers were not required to ask rescuers about any change of the patient, but some dispatchers actually did in this study. Our study pointed out this misunderstanding of the standard protocol. It is important to note that dispatchers should not ask about the patients’ status until the rescuer notices and reports these changes in order to facilitate the performance of continuous chest compressions.

Performing DACRP is not an easy task for both lay rescuers and dispatchers. Lay rescuers may have poor understanding of CPR, even among those who have training experience. For dispatchers, on the other hand, having to deal with audio information, and no visual information can make this a very difficult task. These circumstances make DACPR notoriously difficult. However, exploring better EMS dispatch instruction to support lay rescuers perform CPR with better quality is the key to further improving survival outcomes for sudden CA victims. As our study showed, giving instructions of the actual depth of 5 cm or to push hard may not be appropriate to achieve the optimal depth of chest compressions by lay rescuers. Since they tend to perform shallow chest compressions from the very beginning, dispatchers may need to keep encouraging rescuers to perform “harder, harder, and much harder” compressions.^21^, ^26^ Future studies on modifying the existing protocol including this coaching process will give us more information on improving the quality of DACPR.

There are several limitations inherit in this study. First, the number of study participants is small and further studies with larger numbers of participants are required. Second, we could not recruit many male participants at the shopping mall. It is known that female gender is associated with poor quality of CPR.^18^ While in real life the majority of bystanders are reported to be female,^27^, ^28^ still this point needs to be considered when it comes to generalization of our study findings. Third, this is an observational simulation study and it is unknown how actual lay rescuers behave and perform CPR in real cardiac arrest cases. Also, CPR instruction used in this study was accepted only in Japan. Although the structure of this protocol was based on standard CPR guidelines, our results may not be generalizable to other countries where different protocols are used. Fourth, we did not collect data regarding recoil of chest compressions. Thus, this may have weakened our study findings. Finally, we could not explore the association between abdominal hand placement and shallow compression depth. It is possible that participants placing their hands close to abdomen could have been the cause of inadequate depth of chest compressions. However we did not obtain detailed information on this issue in the current study.

## Conclusions

This current observational simulation study showed that the quality of DACPR by lay rescuers can be less than optimal in terms of compression depth, hand placement and the minimization of compression pauses. Several specific areas of concern were identified as needing further investigation in order to optimize DACPR. Further studies are required to explore these areas and provide better DACPR instruction that will help lay rescuers perform CPR with optimal quality.

## Disclosure

The study protocol was approved by the eithics committee of Nara Medical University.

Written informed consent was obtained from each study participant.

No registration number is available for this study.

Conflicts of interest: Authors have no conflicts of interest to declare.
